# Supplementary material for: Metabolic flexibility via mitochondrial BCAA carrier SLC25A44 is required for optimal fever
Source: eLife. 2021 May 4;10:e66865. doi: 10.7554/eLife.66865 (PMC8137140; doi:10.7554/eLife.66865)
Supplement: Supplementary file 1. [file elife-66865-supp1.docx]

**Supplemental File 1**

**Supplemental Table 1. Primer sequences**

| Gene | Forward primer | Reverse primer |
| --- | --- | --- |
| *Slc25a44* | TCGCTGCTAACGTACATCCC | AGACAATGTGAGGGCACTCC |
| *Serca2b* | ACCTTTGCCGCTCATTTTCCAG | AGGCTGCACACACTCTTTACC |
| *36B4* | GGCCCTGCACTCTCGCTTTC | TGCCAGGACGCGCTTGT |
